# Novel flow cytometry approach to identify bronchial epithelial cells from healthy human airways

**DOI:** 10.1038/srep42214

**Published:** 2017-02-06

**Authors:** Danay Maestre-Batlle, Olga M. Pena, Jeremy A. Hirota, Evelyn Gunawan, Christopher F. Rider, Darren Sutherland, Neil E. Alexis, Chris Carlsten

**Affiliations:** 1 Chan-Yeung Center for Occupational and Environmental Respiratory Disease, Department of Medicine, Division of Respiratory Medicine, University of British Columbia, Vancouver, British Columbia, Canada; 2 Department of Pediatrics, University of North Carolina at Chapel Hill, USA

## Abstract

Sampling various compartments within the lower airways to examine human bronchial epithelial cells (HBEC) is essential for understanding numerous lung diseases. Conventional methods to identify HBEC in bronchoalveolar lavage (BAL) and wash (BW) have throughput limitations in terms of efficiency and ensuring adequate cell numbers for quantification. Flow cytometry can provide high-throughput quantification of cell number and function in BAL and BW samples, while requiring low cell numbers. To date, a flow cytometric method to identify HBEC recovered from lower human airway samples is unavailable. In this study we present a flow cytometric method identifying HBEC as CD45 negative, EpCAM/pan-cytokeratin (pan-CK) double-positive population after excluding debris, doublets and dead cells from the analysis. For validation, the HBEC panel was applied to primary HBEC resulting in 98.6% of live cells. In healthy volunteers, HBEC recovered from BAL (2.3% of live cells), BW (32.5%) and bronchial brushing samples (88.9%) correlated significantly (p = 0.0001) with the manual microscopy counts with an overall Pearson correlation of 0.96 across the three sample types. We therefore have developed, validated, and applied a flow cytometric method that will be useful to interrogate the role of the respiratory epithelium in multiple lung diseases.

The human airway epithelium is the primary impact zone for inhaled environmental factors such as pathogens, allergens, and pollutants^1^,^2^,^3^. It plays an essential role as a protective barrier to the external environment and also mediates immune responses important in antigen presentation and producing inflammatory mediators^4^,^5^,^6^. Evidence suggests that disruptions in the respiratory epithelium may be an underlying mechanistic feature linking air pollution exposure and the development and worsening of respiratory conditions such as asthma^7^,^8^,^9^,^10^,^11^,^12^. Consistent with this epithelium-focused view, studies have connected airway hyperresponsiveness in asthma to the shedding of the bronchial epithelium^13^. For these reasons, bronchial epithelial cells are an important cell type to examine and optimally characterize *in vivo* in humans. Collection of HBEC *in vivo* can be accomplished with BAL (distal airways), BW (proximal airways), and bronchial brushings, where each provides valuable information on the biology of the respiratory epithelium in those distinct airway regions^14^.

Conventional methods to distinguish, quantify and characterize HBEC from other inflammatory and immune cells in lower airway samples include cytochemical staining, immunohistochemical procedures, standard and confocal microscopy and *in-situ* hybridization^15^. These techniques however, have significant limitations in terms of the number of cells quantified, ability to measure cell activation and the substantial time needed to prepare and analyze samples. Flow cytometry is a powerful tool that uses a combination of light scatter properties and cell protein specific antibodies to identify and differentiate specific cell populations as well as assess cell function^16^. Moreover, flow is not subject to the same throughput limitations as conventional methods^17^.

Presently, there is no validated flow cytometric method to identify and optimally characterize HBECs in clinical research samples. Such a method would enable a more detailed interrogation into the role played by the respiratory epithelium in multiple lung diseases. Our goal in this study was to develop, validate and apply a flow cytometric method for the identification and quantification of HBEC from BAL, BW and bronchial brushing samples. Some of the results of this study have been previously reported in the form of an abstract.

## Methods

### Ethics Statement

Human samples were collected from a large parent study approved by the University of British Columbia Clinical Research Ethics Board and informed written consent was obtained from all study participants involved. All experiments were performed in accordance with relevant guidelines and regulations. No deviations were made from our approved protocol (H11-01831).

### Human Samples

BAL, BW and bronchial brushing samples were obtained from participants undergoing a bronchoscopy procedure administered by a respirologist at Vancouver General Hospital as previously described^18^. Sterile saline (0.9% NaCl; Baxter, ON) was instilled through the bronchoscope and almost immediately recovered by applying suction (25–100 mmHg). BW was collected as the return from 2 × 20 ml instilled saline and BAL was subsequently collected as the return from 2 × 50 ml additional saline. Using a bronchial cytology brush (Hobbs Medical Inc, CT) brushings were collected from the endobronchial mucosa of a 4^th^ order airway, similar to but distinct from that used to obtain BAL/BW, and stored in RPMI-1640 (R8748; Sigma, MO) prior to processing.

### Sample Processing

Bronchial brushes were washed approximately 20 times, by pipetting up and down, to remove cells from the brush and collect them in RPMI-1640 media. BAL and BW samples were passed through a 40 μm cell strainer to remove debris and clumped tissue. All 3 lung samples were centrifuged at 300 × g for 10 min at room temperature, low brake. Cell pellets were resuspended in 1 ml of RPMI-1640, manually counted using a hemocytometer, viability was determined by trypan blue exclusion (Gibco, NY) and aliquots were then separated for histology and flow cytometry.

### Submerged and Air-Liquid Interface (ALI) Cultures of Primary Human Bronchial Epithelial Cells (pHBEC)

Cells obtained from bronchial brushes were centrifuged and the pellet resuspended in 1 ml of PneumaCult-Ex medium (STEMCELL Technologies, BC). Following total cell count in an improved Neubauer chamber (mean cell yield = 5 × 10^5^ cells), cells were seeded in a 25 cm^2^ cell culture flask (BioCoat Collagen I; Corning, NY) in 5 ml of PneumaCult-Ex for the expansion of primary human airway cells under submerged culture. Flasks were incubated at 37 °C in 5% CO_2_ until cells were ready to be differentiated and grown at the air-liquid interface. A group of these cells was analyzed by flow cytometry at this stage (submerged culture), while the remaining cells were cultured on 12 mm polyester transwell inserts with a pore size of 0.4 μm (Corning, NY). Cells were plated at a concentration of 100,000 cells per insert in PneumaCult-ALI medium following standard protocol procedures from STEMCELL Technologies. To prepare ALI cells for flow cytometry analysis, media from the wells below the insert was removed and the cells washed with Hanks’ Balanced Salt solution (HBSS) (Sigma-Aldrich, MO) to eliminate all traces of serum and the mucus produced by the cells. HBSS was removed by aspiration followed by the addition of 300 μl/well of 0.5% Trypsin/EDTA (Lonza, NJ) onto the cells. The pseudostratified layer of human bronchial epithelial cells was completely covered and incubated at 37 °C for 5 minutes. The addition of 300 μl/well of Trypsin Neutralizing Solution (Lonza, NJ), was used to effectively neutralize Trypsin/EDTA. Cells were re-suspended by gently pipetting the cell suspension up and down to break up the clumps and centrifuged at 300 × g for 5 minutes. The supernatant was discarded and pelleted cells were promptly stained for flow cytometry.

### Histology

BAL, BW and bronchial brushing cell suspensions were adjusted to 3 × 10^4^ cells in 50 μl of RPMI-1640 and cytocentrifuged at 400 × g for 5 minutes, stained following the May-Grünwald Giemsa (MGG) procedure and coverslips applied with Permount (Thermo Fisher Scientific, MA). Images were captured at 60x magnification on a EVOS FL Auto Imaging System and differential cell counts for percentages of HBEC, macrophages, eosinophils, lymphocytes and neutrophils performed.

### Flow Cytometry

BAL, BW, and bronchial brushings (ranging from 1 × 10^3^ to 40 × 10^3^ cells in 100 μl), and pHBEC both submerged and ALI (30 × 10^3^ to 50 × 10^3^ cells in 100 μl), were used for flow cytometric analysis. Mouse serum (Sigma-Aldrich, MO) was added to the samples and incubated for 15 minutes to block non-specific binding. Cells were stained with antibodies against the surface markers epithelial cell adhesion molecule (EpCAM) (conjugated to a tandem conjugate fluorochrome that combines PerCP with a cyanine dye (PerCP-Cy5.5)) and protein tyrosine phosphate receptor type C (PTPRC; CD45) (conjugated to a tandem fluorochrome that combines APC and a cyanine dye (APC-Cy7)) and incubated for 20 minutes at room temperature in the dark. Fixable viability dye eFluor 450 was added and incubated for 10 minutes, followed by fixation and permeabilization using the FIX & PERM Cell Permeabilization Kit (Invitrogen, MA), and the addition of the intracellular marker pan-cytokeratin.

Anti-pan-cytokeratin antibody recognizes epitopes expressed in the epithelia and trichocytes that are therefore present in most human epithelial tissues^19^. The epithelial cellular adhesion molecule (EpCAM) is expressed exclusively in epithelia^20^, whereas the leukocyte common antigen protein tyrosine phosphatase (CD45), is present in hematopoietic cells, except erythrocytes and platelets^21^.

Table 1 describes the fluorochrome-conjugated antibodies, isotype controls and viability dye used to identify HBEC from lung samples, as well as the supplier, catalog number and quantity added per sample.

Cells were analyzed by flow cytometry using a BD FACSCanto II instrument (BD Biosciences, San Jose, CA) equipped with a coherent sapphire solid state 488-nm (blue) laser, a JDS Uniphase HeNe air cooled 633-nm (red) laser and a Point Source iFLEX2000-P-1-405-0.65-30-NP 405-nm (violet) laser. The BD FACSCanto II 4-2-2 configuration collection optics included one octagon and two trigon detector arrays. Before acquiring the data, 8 point beads were used for manual quality control of the instrument. Unstained cells and compensation beads (BD Biosciences, CA) were used to set voltages and create single stain negative and positive controls. Compensation was set to account for spectral overlap between the four fluorescent channels used in our study. The gating region was set so that less than 1% of the samples stained with negative controls (isotype control and fluorescence minus one (FMO)) fell into the window.

Samples were examined by side scatter area (SSC-A) versus forward scatter area (FSC-A), then using forward scatter height (FSC-H) versus FSC-A to select single cells, eliminating debris and clumped cells from the analysis. Single cells were sub-gated using fixable viability dye eFluor 450 and subsequently live cells were discriminated by the expression of CD45 linked APC-Cy7. Exclusion of CD45 positive cells, a commonly used marker for total leukocytes not expressed on HBEC, was followed by the examination of double expression of pan-cytokeratin FITC intracellular marker and EpCAM PerCP-Cy5.5 cell surface marker. After acquisition, data were exported and analyzed using FlowJo version 10.1r5 (Treestar, OR).

To determine the positive/negative cut-off for the gating strategy in this study, two types of gating controls were used. Isotype controls addressed background due to nonspecific antibody binding, whereas FMO controls were used to detect spillover-induced background^22^. Debris, doublets and dead cells were excluded from the analysis and mouse serum (a commonly used blocker of non-specific binding) was added.

### Statistical Analysis

Mean and standard deviation values were calculated from the total, live and CD45 negative flow cytometry frequencies and the microscopy manual counts using Microsoft Excel 2013. Pearson correlation between microscopy manual counts and flow cytometry frequency of live cells was determined using the ‘rcorr’ function from the Hmisc (v3.17-4) package in R (v 3.3.1).

## Results

### Validating the flow cytometry panel using pHBEC

**Figure 1:**
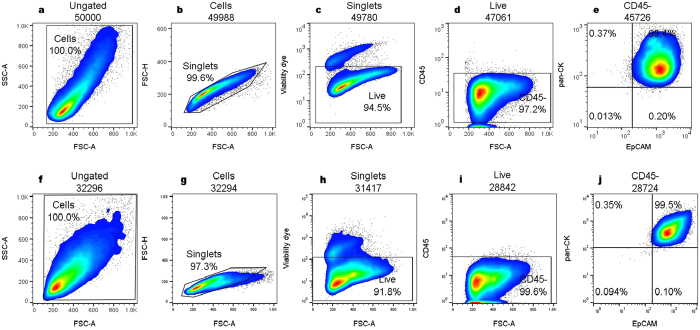
Flow cytometric (pseudo-color/smooth) plots showing the gating strategy validating the identification of HBEC from cultured pHBEC. Cells were gated based on size and granularity using FSC-A vs SSC-A to eliminate debris and clumped cells. Single cells were sub-gated using fixable viability dye eFluor 450 and subsequently live cells were discriminated by the expression of CD45 APC-Cy7. Exclusion of CD45 positive cells was followed by the examination of double expression of pan-cytokeratin FITC and EpCAM PerCP-Cy5.5. Values inside the plots represent the percentages from the parent gate. SSC-A: side scatter area, FSC-A: forward scatter area, FSC-H: forward scatter height. (**a**–**e**) Submerged culture and (**f**–**j)** ALI cultures.

For validation, the HBEC panel was applied to submerged and ALI differentiated pHBEC. Proof of concept for the use of the HBEC antibody panel was determined by staining both stages of the pHBEC culture as a positive control, combining intracellular (pan-CK) and cell surface (EpCAM) antibody markers (Fig. 1). Cells were first gated by size and granularity, selecting a minimum (filter level)/maximum SSC-A/FSC-A to exclude cellular debris. From the “cells” gate, singlets were sub-gated by their FSC-H/FSC-A properties. Single cells (“singlets”) were plotted with the viability dye and those considered “live” were selected (Fig. 1[a–d and f–i]). From the “live” population, those that were CD45- (i.e. not leukocytes) were depicted showing the double expression of pan-cytokeratin (pan-CK) and EpCAM markers (Fig. 1[e and j]). The mean and standard deviation of frequency values observed for the submerged culture (n = 3) was 94.7 ± 2.4% from total cells, 97.9 ± 0.9% from live cells and 99.6 ± 0.2% from CD45- cells. The respective values for ALI cultures were 89.0 ± 3.5% (total cells), 99.3 ± 0.05% (live cells) and 99.6 ± 0.05% (CD45- cells) for n = 2.

### Advantage of dual markers

**Figure 2:**
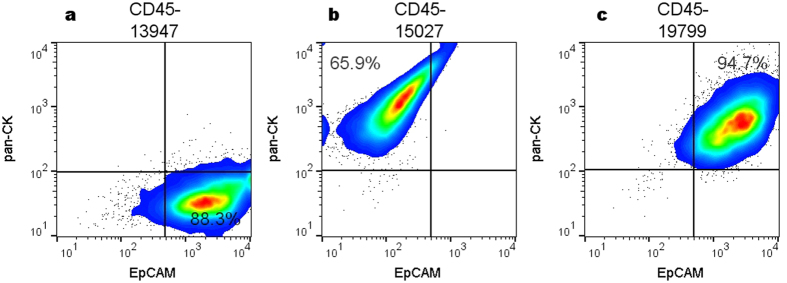
Flow cytometric (pseudo-color/smooth) plots showing cultured pHBEC stained with (**a)** EpCAM only, (**b)** pan-CK only, and (**c)** EpCAM plus pan-CK antibodies. Samples also included the viability dye and CD45 fluorochrome-conjugated antibody. Values inside each plot represent the percentages of HBEC live single cells from a total of approximately 2 × 10^4^ cells acquired.

To determine the value of having dual markers, EpCAM (surface) and pan-CK (intracellular), as part of the staining panel, a comparison between cultured pHBEC populations stained with both (EpCAM and pan-CK) markers versus those stained with only one (EpCAM or pan-CK) was made (Fig. 2). This demonstrates how the inclusion of both markers (Fig. 2c) identified 95% of live HBEC, whereas the use of only EpCAM (Fig. 2a) or pan-CK (Fig. 2b) recognized 88% and 66% respectively.

### Assessing non-specific binding

**Figure 3:**
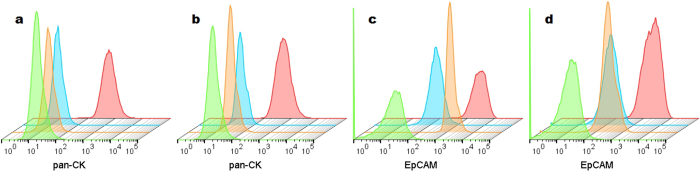
Flow cytometric staggered histograms of fully stained cultured pHBEC (red) and negative controls: unstained sample (green), isotype control (orange) and fluorescence minus-one (FMO) controls (blue). N = 1 per condition. All plots were gated on single, viable, CD45 negative cells. (**a**,**c**) without blocker. (**b**,**d**) with blocker (mouse serum).

Unstained, isotype and fluorescence minus one (FMO) controls were included to set gate limits and determine if any non-specific binding of the panel antibodies to the cellular surface occurred (Fig. 3). The addition of mouse serum had an evident effect when histograms with and without blocker were compared, particularly in the case of EpCAM surface marker. This suggests that the addition of mouse serum is necessary for the adequate and accurate characterization of HBEC when using the proposed panel.

### Applying the flow panel to lung samples

**Figure 4:**
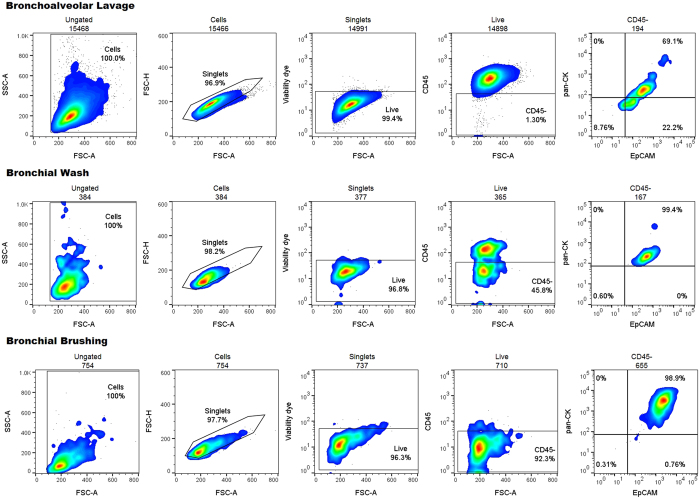
Flow cytometric (pseudo-color/smooth) plots showing the HBEC gating strategy and percentages from the respective parent gate of human BAL, BW and bronchial brushing samples.

BAL, BW and bronchial brushing samples from an ongoing clinical study were stained using the HBEC antibody panel and flow cytometry was performed (Fig. 4). Donor characteristics can be observed in Table 2. Samples from 1, 3 and 4 come from the same donor on different dates. As expected, BAL and BW contained a lower proportion of HBECs (2.3 ± 2.4% and 32.5 ± 24.0% respectively, when compared to bronchial brushings (88.9 ± 4.9%); Table 3). These values represent the percentages of HBEC after debris, doublets and dead cells were excluded which is the equivalent to the percentage of live, single, CD45-negative HBECs. These results indicate that the flow cytometric panel was reliable with a well-defined EpCAM and pan-CK double positive population identified for all samples.

### Comparing to conventional histochemistry

**Figure 5:**
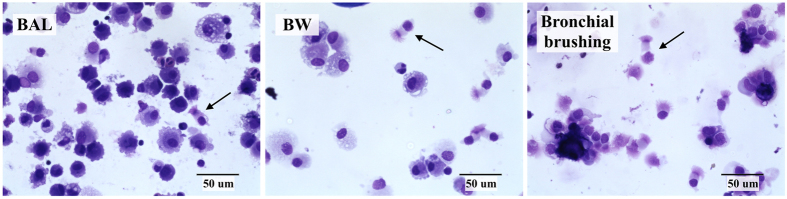
May-Grünwald Giemsa-stained BAL, BW and bronchial brushing samples showing HBEC. 3 × 10^4^ cells were cytocentrifuged and fixed onto slides by dipping in methanol, once dry; fixed cells were stained with methylene blue and eosin solutions followed by a rinse with deionized water. A drop of Permount was added onto the slides, coverslips applied and air-dried for 24 hours until ready to be analyzed. Arrows are indicating examples of HBEC.

To compare the flow cytometry panel to conventional histochemistry, samples were spun onto cytospin slides, May-Grünwald Giemsa (MGG) stained and visualized under light microscopy (Fig. 5), with ciliated columnar epithelial cells identified by their distinctive morphological characteristics. The determination of HBEC cells obtained by flow cytometric analysis of lower human airway samples was consistent with manual counts of Giemsa-stained samples (Table 3), and with the literature on immunohistochemical and cytochemical staining counts^23^,^24^.

**Figure 6:**
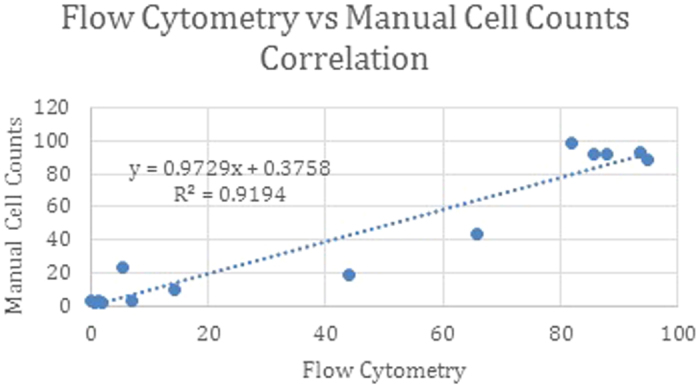
Correlation between manual cell counts and our flow cytometry panel. Cell counts and flow values were plotted across the three sample types (Brushings, BW, BAL). A line of best fit was calculated and the R^2^ value determined. Correlation is the square root of the R^2^ value which equals 0.96.

Additionally, we obtained a strong concordance between the manual microscopy counts and frequency of live HBEC cells identified by flow cytometry, with an overall Pearson correlation of 0.96 and p-value of 0.0001, across the three sample types (Fig. 6).

## Discussion

This study aimed to satisfy the existing need for a validated flow cytometric method for the identification of HBEC from human clinical lung samples. We developed a fluorochrome-conjugated antibody staining panel combining intracellular and cell surface markers and validated it with the use of pHBEC. ALI culture of pHBECs is increasingly being recognized as an important culture system that facilitates physiologically relevant respiratory research as cells grown in the ALI system undergo extensive mucociliary differentiation, resulting in an *in vitro* model that is representative of the *in vivo* airway^25^. The panel was applied to both submerged and ALI differentiated pHBEC as a positive control and to lung samples (BAL, BW and bronchial brushings) containing various percentages of HBEC. A significant correlation was observed when the frequency values of live cells obtained by flow cytometry were compared to the conventional method of manual counts of Giemsa-stained slides. We therefore have developed, validated, and applied a flow cytometric method that will be useful to extend interrogations into the role the respiratory epithelium plays in multiple lung diseases.

Efforts to study a number of pulmonary diseases, particularly those related to airway infection, oxidative stress, inflammation and potential treatments relevant to lung injuries, often rely on conventional methods for the identification of HBEC^26^,^27^,^28^,^29^. These methods include cytochemical and immuno-histochemical staining, *in-situ* hybridization, standard and confocal microscopy^13^,^30^,^31^. The ability to detect antigens in tissue sections has improved dramatically in the past years^32^, but these conventional methods have noticeable disadvantages regarding the ability to simultaneously analyze activation states in multiple cell types, the small number of cells being studied and temporal efficiency, so they remain time-consuming and without the potential for high-throughput analysis^33^,^34^. Therefore, clinically focused discoveries involving HBEC responses, such as expression of cytokines, chemokines, surface receptors, binding proteins and activation of signalling pathways require a more comprehensive characterization method, which is currently lacking^35^,^36^. Conversely, flow cytometry allows observation and characterisation of numerous stimulation-dependent changes including modulation of proliferation, crosslinking of membrane glycoproteins, altered expression of cell surface adhesion molecules and protein expression^37^,^38^. Furthermore, quantitative cytology in the form of flow cytometry has greatly advanced the field of clinical research, with new antibodies, improved gating strategies and routine use of multi-parameter techniques dramatically improving diagnostic utility^39^,^40^. Our validated flow cytometric method is a strong addition to conventional methods to study pulmonary diseases where HBEC are believed to play a contributing role in exacerbations and pathology.

The present study with clinical samples expands on our historical application of flow cytometry for analyzing leukocytes and phagocytes from human BAL, bronchial brushing and induced sputum^41^,^42^. In this study, we processed, stained and acquired data within 2 hours of collecting the sample after bronchoscopy. We were able to analyze samples from different areas of the lung that contained different percentages of HBEC. The sample types collected provide a representation of respiratory epithelial cell populations throughout the lung. Our data suggest that irrespective of the sample type collected, application of the flow cytometric method will yield analysis of the population of epithelial cells present. Our data confirm that the purity of epithelial cells in a given clinical sample may vary depending on the anatomical location it was collected from. Bronchial brushings are primarily composed of HBEC and this sample may be most useful in providing a detailed examination of epithelial function. Cell yields from bronchial brushings often vary, as samples typically contain cellular debris due to the physical disruption of the airway epithelium during sample collection. Bronchial washes sampled the proximal airways and contained an intermediate percentage of HBEC. Bronchial lavages sampled the more distal airways (in comparison to BW), and produced an increasingly mixed cell sample containing a low but still distinguishable percentage of HBEC^23^. Collectively, our data validate a flow cytometric method for identifying HBEC, applicable to a variety of clinical samples from different anatomical locations within the lung, that can complement more common analysis of immune cells.

Manual microscopy-based analysis of immune-histochemical and cytochemical-stained samples remain the current gold standard approach to quantify and characterize HBEC in clinical samples^43^,^44^. We identified a high degree of correlation between percentages of HBEC from BAL, BW and bronchial brushings, in manual counts from microscopy versus the frequency of live (single, CD45 negative, EpCAM/pan-CK double positive) cells after flow cytometric analysis, suggesting that these methods are comparable. Likewise, values for epithelial cell percentages for all three sample types analysed were comparable to the expected values from the literature, when conventional cytochemical methods were used^24^. The flow cytometric method proposed in this study meets and for several applications exceeds conventional techniques for the identification of HBEC from human lung samples.

Our study was done on a three laser and eight fluorochrome channel instrument where only four channels were being used. Modern flow cytometry methods frequently include over ten fluorochromes, allowing a great potential of expanding the proposed panel^45^,^46^. Establishing a fluorescence based cytometry panel could provide a foundation for transition into flow RNA assays or mass cytometry with simultaneous analysis of immune cells^47^. Furthermore, mass cytometry technology, which uses antibodies conjugated to isotopically pure rare earth elements (eliminating the potential spectral overlap from the fluorochromes emission signal), could be added to the analysis of HBEC, expanding the data obtainable from a single sample^48^,^49^.

In summary, we have developed, validated, and applied a reliable and reproducible flow cytometry acquisition and analysis method for identifying HBEC. This approach avoids the limitations inherent in conventional analysis techniques, expanding capabilities in further elucidating the role of HBEC in health and disease. In particular, our methodological strategy can provide valuable insight into the immune responses of respiratory epithelium in response to the external environment relevant in chronic lung diseases including asthma, COPD, cystic fibrosis, and pulmonary fibrosis. These studies could identify biomarkers of exposure effect, supporting prevention, or suggest therapeutic targets of immune dysfunction of the epithelium present in respiratory diseases.

## Additional Information

**How to cite this article**: Maestre-Batlle, D. *et al*. Novel flow cytometry approach to identify bronchial epithelial cells from healthy human airways. *Sci. Rep.* **7**, 42214; doi: 10.1038/srep42214 (2017).

**Publisher's note:** Springer Nature remains neutral with regard to jurisdictional claims in published maps and institutional affiliations.

## Figures and Tables

**Table 1:** Fluorochrome-conjugated antibodies, isotype controls and viability dye used to identify HBEC from lung samples.

Conjugated antibody [Clone]	Supplier	Catalog Number	Quantity per sample
*pan-cytokeratin FITC [C-11]*	Abcam	ab78478*	3 μl
*EpCAM PerCP-Cy5.5* [*EBA-1*]	BD Biosciences	347199	3 μl
*CD45 APC-Cy7* [*2D1*]	BD Biosciences	557833	3 μl
*FITC mouse IgG1 (Isotype control*)	BD Biosciences	555748	3 μl
*PerCP-Cy5.5 mouse IgG1 (Isotype control*)	BD Biosciences	550795	3 μl
*Fixable Viability Dye eFluor 450*	eBioscience	65-0863-14	2 μl

Supplier information, catalog number and quantity added per sample also included. *No longer available; has been replaced by ab11214.

**Table 2:** Donor characteristics.

	*Donor Characteristics*				
*N*	1	2	3	4	5
*Gender*	Male	Female	Male	Male	Male
*Age*	28	23	28	28	65
*Health Status*	Mild Asthmatic				Mild COPD

**Table 3:** HBEC as portion of cells present in lung samples according to flow cytometry, versus manual count using light microscopy after Giemsa-staining.

	Percent of Total	Percent of CD45-	Percent of Live	Percent by microscopy
*BAL (n = 5*)	1.9 ± 1.9	45.3 ± 31.2	2.3 ± 2.4	3.1 ± 0.6
*BW (n = 4*)	27.9 ± 20.5	70.5 ± 22.2	32.5 ± 24.0	26.6 ± 12.2
*Brushing (n = 5*)	76.1 ± 11.0	92.9 ± 5.8	88.9 ± 4.9	92.7 ± 3.2
*Submerged pHBEC (n = 3*)	94.7 ± 2.4	99.6 ± 0.2	97.9 ± 0.9	
*ALI pHBEC (n = 2*)	89.0 ± 3.5	99.6 ± 0.05	99.3 ± 0.05	

Percentages of positive control pHBEC (submerged and ALI) obtained by flow cytometry are also presented.
